# Low-Intensity Vibration Improves Muscle Healing in a Mouse Model of Laceration Injury

**DOI:** 10.3390/jfmk3010001

**Published:** 2017-12-21

**Authors:** Thomas F. Corbiere, Eileen M. Weinheimer-Haus, Stefan Judex, Timothy J. Koh

**Affiliations:** 1 Department of Kinesiology and Nutrition, University of Illinois at Chicago, Chicago, IL 60612, USA; 2 Center for Wound Healing and Tissue Regeneration, University of Illinois at Chicago, Chicago, IL 60612, USA; 3 Department of Biomedical Engineering, Stony Brook University, Stony Brook, NY 11794, USA

**Keywords:** skeletal muscle injury, laceration, low-intensity vibration, muscle regeneration, fibrosis

## Abstract

Recovery from traumatic muscle injuries is typically prolonged and incomplete, leading to impaired muscle and joint function. We sought to determine whether mechanical stimulation via whole-body low-intensity vibration (LIV) could (1) improve muscle regeneration and (2) reduce muscle fibrosis following traumatic injury. C57BL/6J mice were subjected to a laceration of the gastrocnemius muscle and were treated with LIV (0.2 g at 90 Hz or 0.4 g at 45 Hz for 30 min/day) or non-LIV sham treatment (controls) for seven or 14 days. Muscle regeneration and fibrosis were assessed in hematoxylin and eosin or Masson’s trichrome stained muscle cryosections, respectively. Compared to non-LIV control mice, the myofiber cross-sectional area was larger in mice treated with each LIV protocol after 14 days of treatment. Minimum fiber diameter was also larger in mice treated with LIV of 90 Hz/0.2 g after 14 days of treatment. There was also a trend toward a reduction in collagen deposition after 14 days of treatment with 45 Hz/0.4 g (*p* = 0.059). These findings suggest that LIV may improve muscle healing by enhancing myofiber growth and reducing fibrosis. The LIV-induced improvements in muscle healing suggest that LIV may represent a novel therapeutic approach for improving the healing of traumatic muscle injuries.

## 1. Introduction

Traumatic muscle injuries are among the most common injuries experienced during military combat. Approximately 70% of combat injuries involve the musculoskeletal system, many of which in recent conflicts have been caused by improvised explosive devices that cause devastating soft tissue injury [1]. Recovery is typically prolonged and incomplete and the inadequate healing response is associated with impaired muscle function, joint stiffness, and loss of mobility [2–5]. The impaired healing of traumatic muscle injuries is likely due, in part, to a disruption in blood supply and subsequent ischemia and development of fibrosis. Current therapies include anti-inflammatory strategies and physical therapy. However, we and others have demonstrated that blocking components of the inflammatory response can lead to impaired muscle healing and reduced muscle growth [4, 6–11]. In addition, physical rehabilitation in the form of voluntary wheel running has resulted in modest functional improvements and increases in muscle mass associated with the upregulation of markers of fibrosis, but no hypertrophy or hyperplasia of muscle fibers after eight weeks [12]. This indicates that the improvements in function may be due to “functional fibrosis”. The lack of significant improvements in muscle function resulting from existing therapeutic approaches indicates that additional therapies are needed.

Skeletal muscle is remarkably sensitive to changes in mechanical loading. Resistance exercise and other forms of mechanical loading increase muscle mass, while reduced loading by immobilization or microgravity leads to muscle atrophy [13–15]. Mechanical stimulation via low-intensity vibration (LIV), defined as vibration with a magnitude less than 1g acceleration, can be considered a physical rehabilitation modality. Whole body mechanical stimulation via LIV has been shown to increase bone and muscle mass in growing mice and to attenuate the loss of bone and muscle during reduced loading situations [16–18]. Furthermore, LIV has been shown to accelerate bone regeneration in a cranial defect in rats [19]. With respect to tissue repair, mechanical stimulation via negative pressure therapy is commonly used to improve skin wound healing, including combat-related blast injuries [20], and we have recently shown that LIV improves the delayed healing of skin wounds in diabetic mice [21]. However, little is known about the influence of any type of mechanical stimulation on the healing of damaged muscle.

We therefore sought to determine whether mechanical stimulation via LIV could improve muscle healing following traumatic injury. We hypothesized that LIV would (1) improve muscle regeneration and (2) reduce muscle fibrosis following traumatic injury in mice. In this manuscript, we present our initial results bearing on these hypotheses.

## 2. Materials and Methods

### 2.1. Animals

C57BL/6J mice were obtained from Jackson Laboratories and housed individually in a pathogen-free, barrier facility with a 12 h light/dark cycle at a constant temperature and humidity. Experiments were performed on male mice 11–13 weeks old. Following traumatic injury of the gastrocnemius muscles, mice were randomly assigned to one of three LIV treatment groups (90 Hz, 14 days treatment; *n* = 14 mice, 45 Hz, 14 days treatment; *n* = 18 mice, 90 Hz 7 days treatment; *n* = 6 mice,) or non-LIV control (*n* = 16, *n* = 18, and *n* = 6 mice, respectively) treatment, starting on the day of wounding. Uninjured control mice were also subjected to the LIV protocol (*n* = 3–5 mice). All procedures involving animals were approved by the Animal Care Committee (Protocol 17-067) at the University of Illinois at Chicago (9 June 2017).

### 2.2. Muscle Injury

Bilateral laceration of the gastrocnemius muscles was used as a model of traumatic injury and was performed as previously described [22]. Briefly, mice were anesthetized and a longitudinal incision was made through the skin on the posterior hindlimb. A scalpel was used to lacerate the lateral gastrocnemius transversely at its widest point, from the central neurovascular complex (taking care to preserve its integrity) to the lateral edge of the muscle, which is approximately 4 mm. The laceration goes through the entire thickness of the mid-belly of the muscle which is approximately 2–3 mm thick. The skin was closed, and the procedure was repeated on the contralateral leg. Muscles from injured and non-injured control mice were harvested at the indicated time points.

### 2.3. Whole Body Low-Intensity Vibration

**Figure 1:**
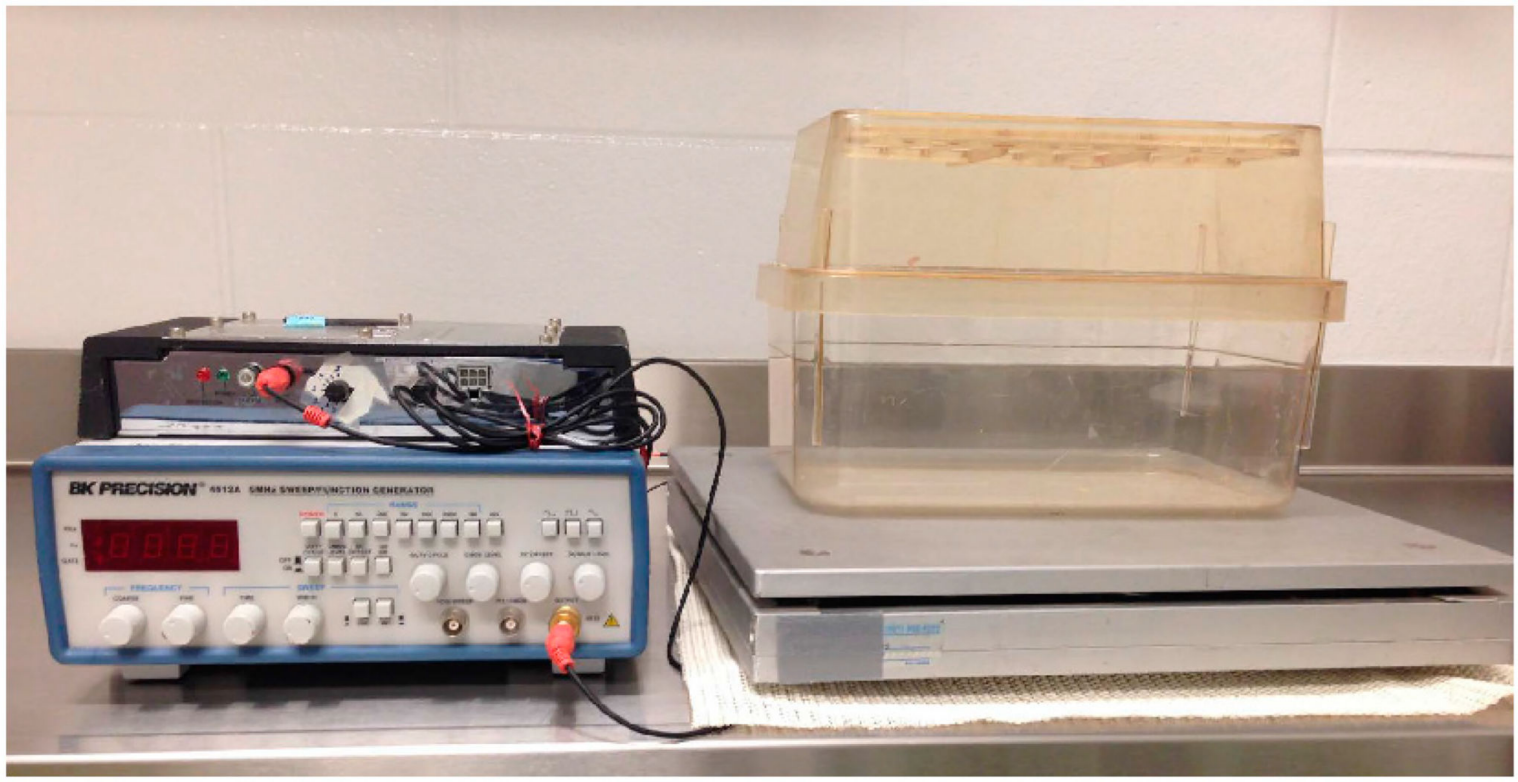
Equipment used to deliver whole-body low-intensity vibration (LIV) to mice. Mice were placed in an empty cage directly on the vibrating plate, and LIV was applied vertically at either 45 Hz or 90 Hz with a peak acceleration of either 0.4 g or 0.2 g for 30 min/day. The non-vibrated controls were similarly placed in a separate empty cage but were not subjected to LIV.

For LIV, mice were placed in an empty cage directly on the vibrating plate and LIV was applied vertically at either 90 Hz with a peak acceleration of 0.2 g or 45 Hz with a peak acceleration of 0.4 g for 30 min per day for either seven or 14 days (Figure 1) [21]. Non-vibrated controls were placed in a separate empty cage but were not subjected to LIV. The mechanical signals were calibrated using an accelerometer attached to the inside surface of the bottom of the cage, so that the signals produced were indeed those transmitted to the feet of the mice. In addition, the amplitude of the vibrations (<100 µm) were small enough that the cage did not move relative to the plate and the vibrations of the plate and the cage were in sync. The protocols used for this study were chosen based on their ability to induce positive biological effects in animals. The 90 Hz/0.2 g protocol has been used to ameliorate bone loss in rodents [23]. The 45 Hz/0.4 g protocol has been used to accelerate bone regeneration in a cranial defect and improve wound healing in rodents [19, 21].

### 2.4. Histology

Muscle regeneration and fibrosis were assessed by histological analysis, as previously described [22]. Gastrocnemius muscles were harvested, embedded in freezing medium, and flash frozen in 2-methylbutane cooled on dry ice. Serial transverse 10 µm-thick cryosections were taken throughout the entire injured portion of the muscle. Sections with the greatest percentage of damaged, non-regenerated area were then selected for further analysis by staining with hematoxylin and eosin and Masson’s trichrome, as well as immunohistochemistry.

Regeneration was quantified in hematoxylin and eosin-stained sections by morphological analysis on five representative images of each muscle section obtained using a Nikon Instruments Eclipse 80i microscope with a 40× objective, a DS-Fi1 digital camera, and NIS Elements software (Nikon, Melville, NY, USA). Images were taken within the muscle belly and care was taken to avoid extramuscular connective tissue. Fibers were identified as either centrally-nucleated or peripherally-nucleated with no evidence of damage. Centrally-nucleated fibers likely represent both fibers that have undergone denervation and those in the process of regeneration [24]. Percent of total fibers that were classified as centrally- or peripherally-nucleated were then quantified using ImageJ (NIH, Bethesda, MD, USA). The damaged area was quantified by subtracting the area of all fibers from the total area within the field of view.

Collagen accumulation was quantified using Masson’s trichrome staining. Three to six 20× images were taken of the injured site in each muscle using a 20× objective on an Eclipse 80i microscope with DS-Fi1 camera and NIS-Elements BR software. Masson’s trichrome stains muscle fibers red, nuclei black, and collagen blue. Collagen accumulation was quantified as the percent of the total image area stained blue.

Platelet endothelial cell adhesion molecule-1 (PECAM-1), a marker for angiogenesis, was identified using anti-mouse CD31 antibody (clone 390; 1:100 in PBS; BioLegend, Inc., San Diego, CA, USA); whereas macrophage accumulation was assessed using an anti-mouse F4/80 antibody (clone BM8; 1:100 in PBS; eBioscience, Inc., San Diego, CA, USA). Slides serving as negative controls received PBS instead of primary antibody. Briefly, sections were air-dried, fixed in cold acetone, washed with PBS, quenched with 0.3% hydrogen peroxide, and washed with PBS. Sections were blocked with buffer containing 3% bovine serum albumin and then incubated with primary antibody for 1 h at room temperature and then overnight at 4 °C. Sections were washed in PBS and incubated with biotinylated mouse adsorbed anti-rat IgG (1:200 in PBS; Vector Laboratories Inc., Burlingame, CA, USA), followed by avid in D horseradish peroxidase (1:1000 in PBS). Sections were then developed with 3,3′-diaminobenzidine (ImmPACT DAB, Cat. No. SK-4105; Vector). Three to six 20× images were taken of the injured site in each muscle using a 20× objective on an Eclipse 80i microscope with DS-Fi1 camera and NIS-Elements BR software. Angiogenesis and macrophage accumulation were quantified as the percent of the total image area stained using ImageJ (NIH).

### 2.5. Statistics

Values are reported as means ± standard error. Data were tested for homoscedasticity and those passed were compared using two-sided *t* tests and those that did not pass were compared using the nonparametric Mann-Whitney test. Differences between groups were considered significant if *p* ≤ 0.05. Graphpad Prism Version 7.00 (Graphpad Software, Inc, San Diego, CA, USA) was used to generate all figures.

### 3. Results

#### 3.1. LIV Protocol Using 90 Hz and 0.2 g for 14 Day Treatment Period

**Figure 2:**
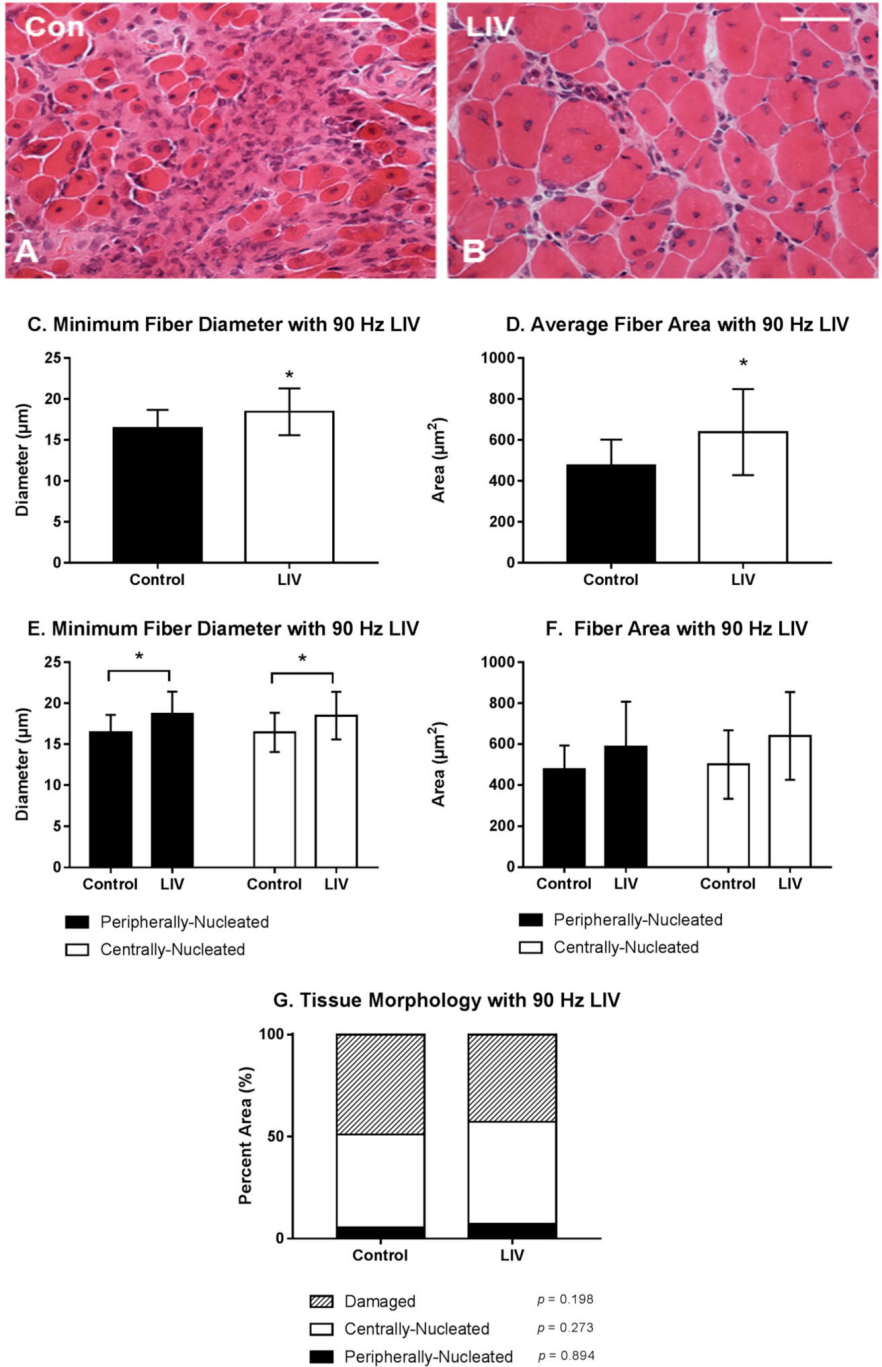
Low-intensity vibration (LIV) at 90 Hz and 0.2 g enhances myofiber growth at day 14 post-injury following laceration muscle injury in mice. Gastrocnemius muscles were lacerated and collected for histological analysis at day 14 post-injury. (**A,B**) Representative images of hematoxylin and eosin-stained sections (scale bar = 50 µm, 40× magnification); (**C,E**) Average minimum myofiber diameter; (**D,F**) average cross-sectional area of individual myofibers, and (**G**) percent area of injury that consists of peripherally-nucleated fibers, centrally-nucleated fibers, or damaged tissue was quantified in five 40× fields per muscle in hematoxylin and eosin-stained sections. (**C,D**) All fiber types averaged together; (**E,F**) Myofibers grouped by type. Data are presented as mean ± SE. * *p* ≤ 0.05.

Body mass (27.3 ± 1.3 g vs. 27.1 ± 1.1 g; *p* > 0.05) was not different between LIV and non-LIV groups, suggesting that mice tolerated the LIV protocol well. Consistent with our hypothesis, LIV treatment at 90 Hz/0.2 g improved the healing of lacerated gastrocnemius muscle at day 14 post-injury (Figure 2A,B). Compared to non-LIV but injured control mice, both the minimum fiber diameter and the cross-sectional area of individual myofibers were significantly larger in mice treated with LIV at 14 days post-injury (Figure 2C,D).When centrally-nucleated and peripherally-nucleated myofibers were assessed separately, the minimum fiber diameters of both were significantly larger in LIV treated mice (Figure 2E), whereas cross-sectional area showed only a trend in this direction (Figure 2F). In contrast to the increase in muscle fiber size, the percent area occupied by centrally-nucleated myofibers and peripherally-nucleated myofibers was not different between LIV-treated and non-LIV control mice; however, there may be a trend towards a decrease in damaged area with LIV (*p* = 0.198) (Figure 2G).

### 3.2. LIV Protocol Using 45 Hz and 0.4 g for 14 Day Treatment Period

**Figure 3:**
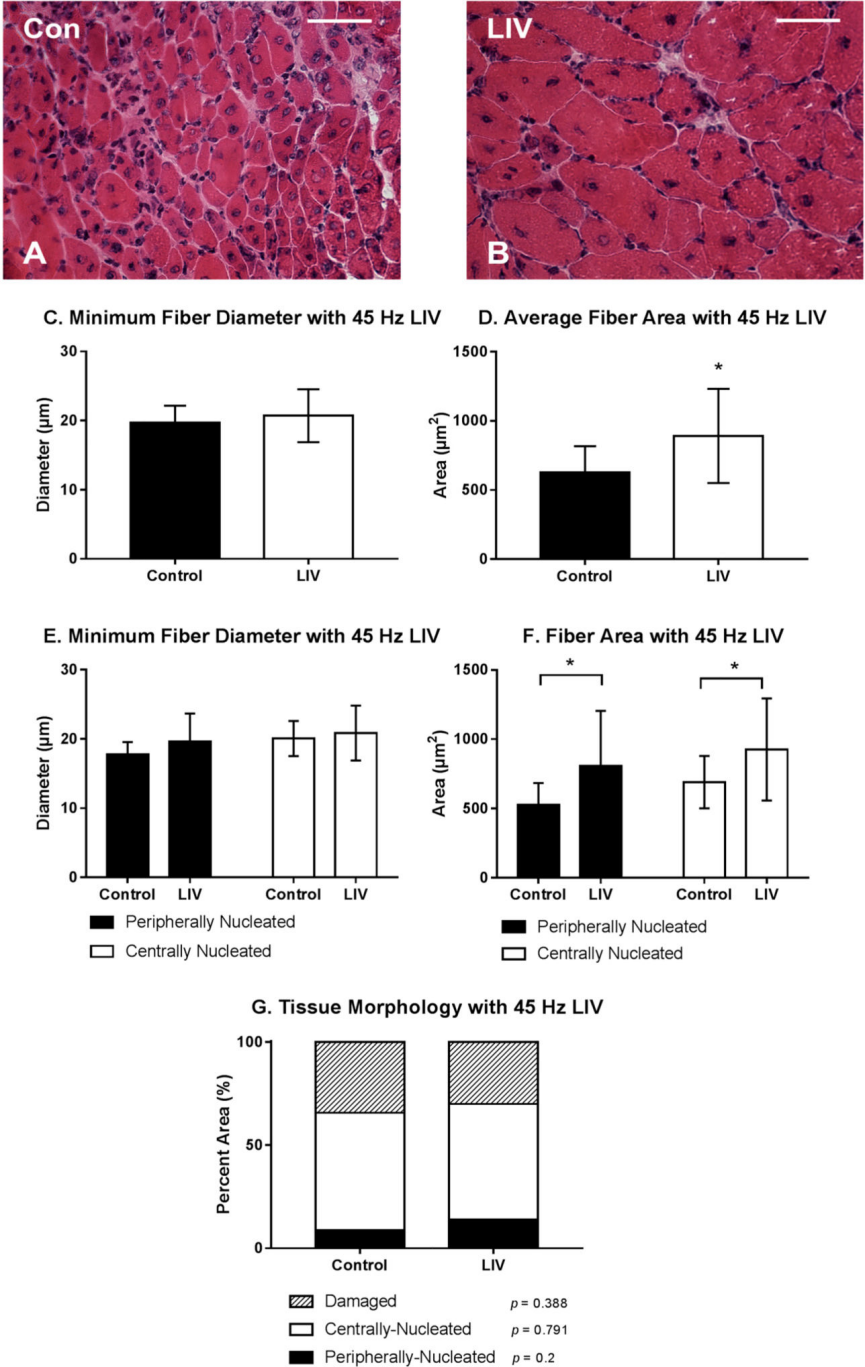
Low-intensity vibration (LIV) at 45 Hz and 0.4 g enhances myofiber growth following laceration muscle injury in mice at 14 days post-injury. Gastrocnemius muscles were lacerated and collected for histological analysis at day 14 post-injury. (**A,B**) Representative images of hematoxylin and eosin-stained sections (scale bar = 50 µm, 40× magnification); (**C,E**) Average minimum myofiber diameter; (**D,F**) average cross-sectional area of individual myofibers, and (**G**) percent area of injury that consists of peripherally-nucleated fibers, centrally-nucleated fibers, or damaged tissue was quantified in five 40× fields per muscle in hematoxylin and eosin-stained sections; (**C,D**) All fiber types averaged together; (**E,F**) Myofibers grouped by type. Data are presented as mean ± SE. * *p* ≤ 0.05.

LIV treatments at 45 Hz/0.4 g also improved the healing of lacerated gastrocnemius muscle (Figure 3A,B). Compared to non-LIV but injured control mice, the cross-sectional area but not the minimum fiber diameter of individual myofibers was larger in mice treated with LIV at 14 days post-injury (Figure 3C,D). When centrally-nucleated and peripherally-nucleated myofibers were assessed separately, the cross-sectional area of both but not the minimum fiber diameter was significantly larger in LIV treated mice (Figure 3E,F). There was no significant difference in the percent area occupied by centrally-nucleated myofibers or damaged area between LIV-treated and non-LIV control mice; however, there may be a trend towards an increase in percent area of peripherally-nucleated fibers with LIV (*p* = 0.2) (Figure 3G). Considering the increases in fiber diameter and/or area between each of the LIV protocols, these morphological data suggest that LIV may not influence the formation of regenerating fibers, but instead enhances myofiber growth after formation.

### 3.3. Effects of LIV on Fibrosis for 14 Day Treatment Period

**Figure 4:**
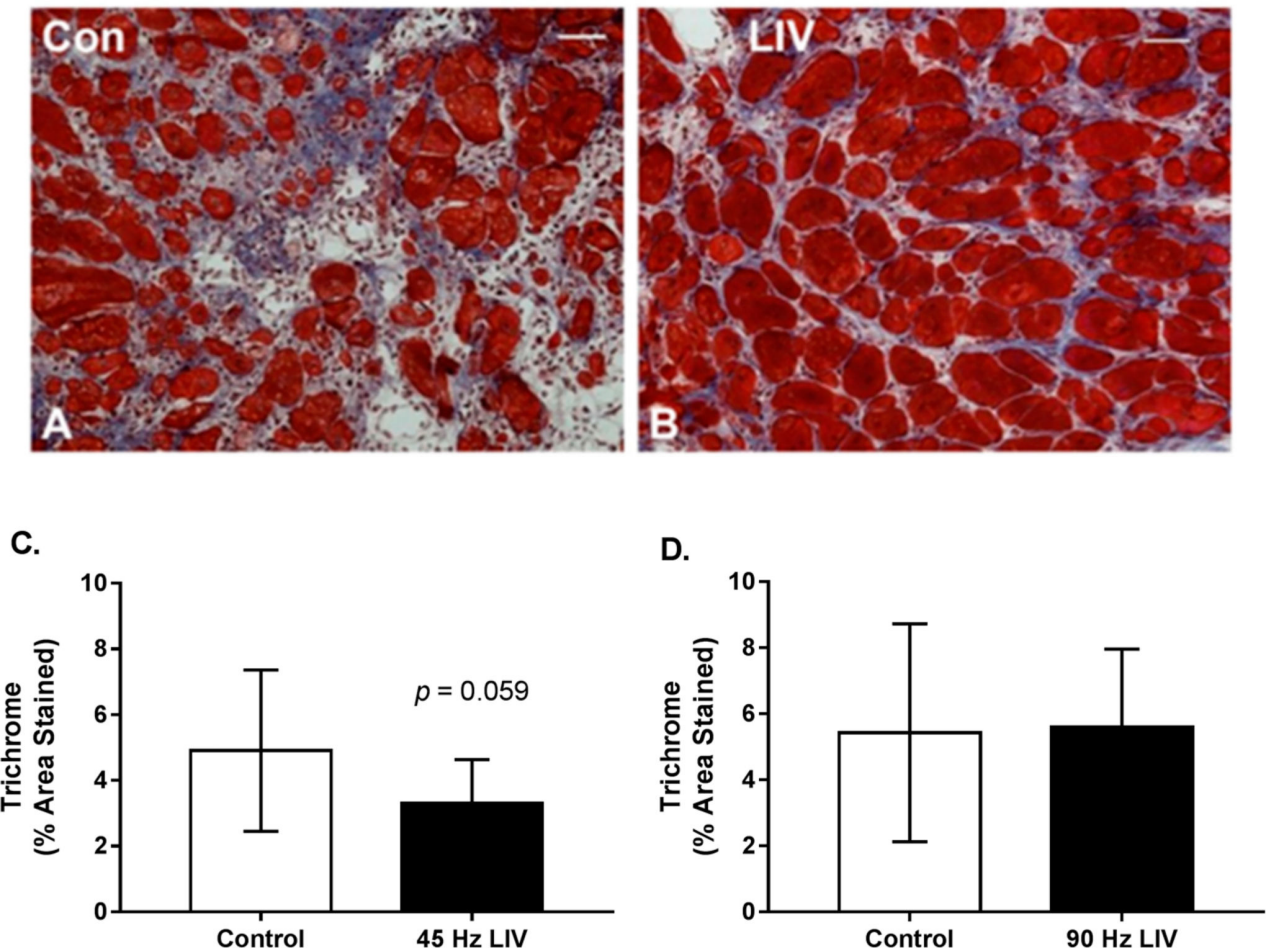
Fibrosis may be reduced in lacerated muscle following low-intensity vibration. (**A,B**) Representative images of trichrome-stained sections at day 14 following laceration of the gastrocnemius muscles (scale bar = 50 µm, 20× magnification); (**C,D**) Collagen accumulation was quantified as percent blue pixels in three to six 20× fields per muscle in Masson’s trichrome-stained sections. Data are presented as mean ± SE. * *p* ≤ 0.05.

Since lacerated gastrocnemius muscle heals by a combination of regeneration and fibrosis, we also assessed the effects of LIV on muscle fibrosis. Trichrome staining in muscle cryosections revealed a trend of reduced collagen deposition in mice treated with 45 Hz/0.4 g LIV vs. non-LIV controls on day 14 following injury. This same effect was not replicated with the 90 Hz/0.2 g LIV protocol (Figure 4A–C). When considered alongside the LIV-induced increase in myofiber cross-sectional area, these findings suggest that LIV, at least the 45 Hz protocol, may improve muscle healing by enhancing myofiber growth and reducing fibrosis.

### 3.4. LIV Protocol Using 90 Hz and 0.2 g for Seven Day Treatment Period

**Figure 5:**
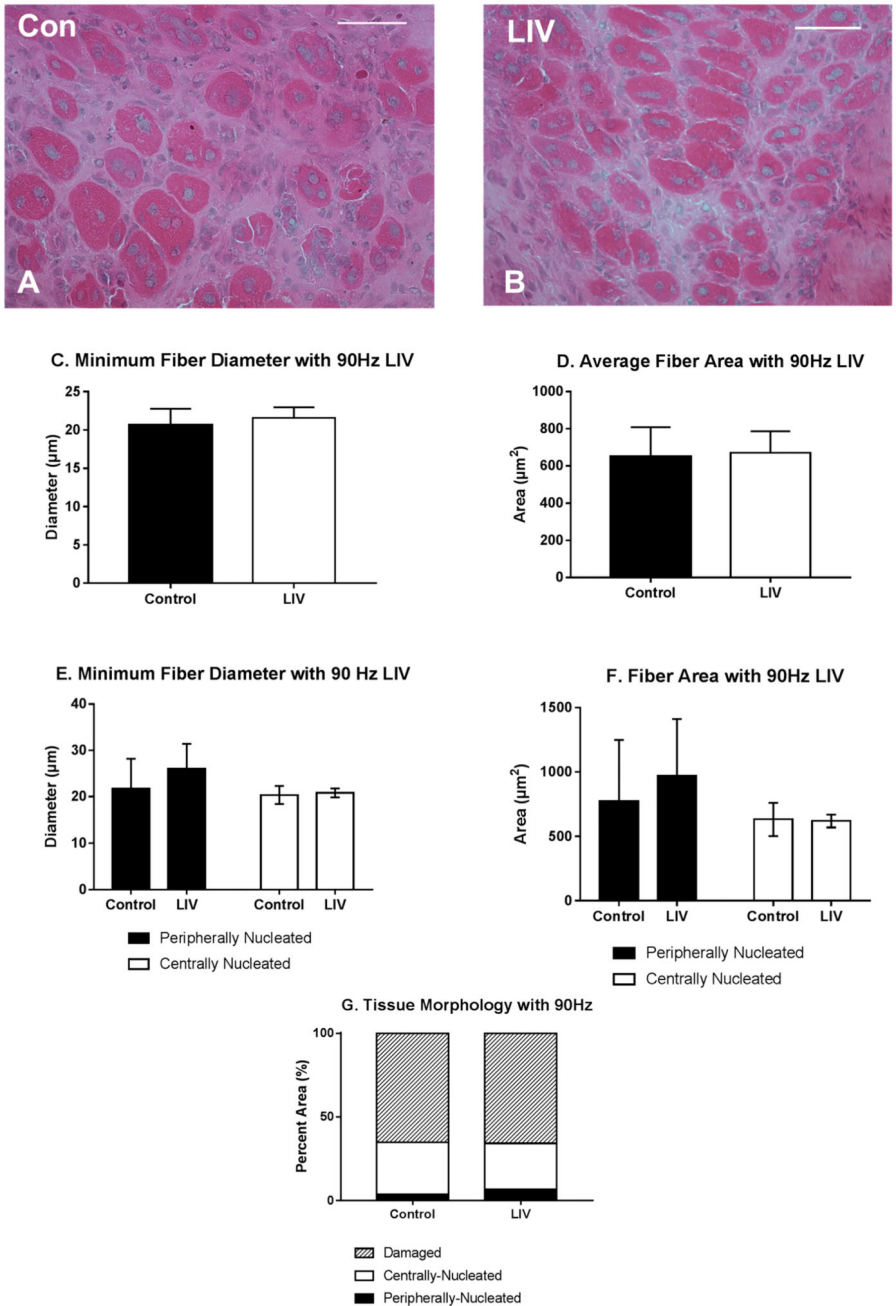
Low-intensity vibration (LIV) at 90 Hz and 0.2 g does not influence muscle regeneration on day seven post-injury following laceration muscle injury in mice. Gastrocnemius muscles were lacerated and collected for histological analysis at day seven post-injury. (**A,B**) Representative images of hematoxylin and eosin-stained sections (scale bar = 50 µm, 40× magnification); (**C,E**) Average minimum myofiber diameter; (**D,F**) average cross-sectional area of individual myofibers, and (**G**) percent area of injury that consists of peripherally-nucleated fibers, centrally-nucleated fibers, or damaged tissue was quantified in five 40× fields per muscle in hematoxylin and eosin-stained sections; (**C,D**) All fiber types averaged together; (**E,F**) Myofibers grouped by type. Data are presented as mean ± SE.

**Figure 6:**
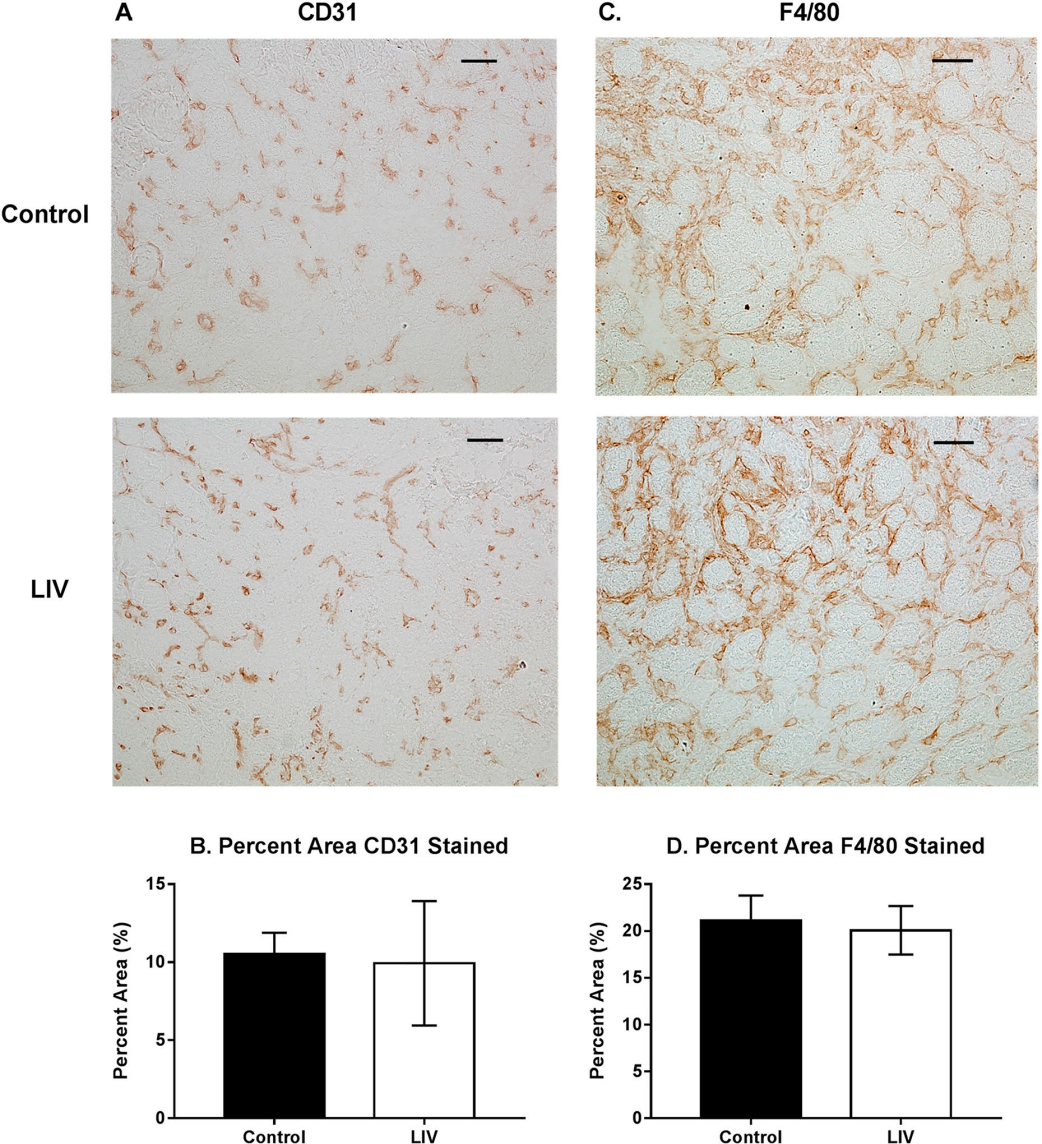
Effects of low-intensity vibration (LIV) at 90 Hz and 0.2 g on angiogenesis (CD31) and macrophage accumulation (F4/80) at day seven post-injury following laceration muscle injury in mice. Gastrocnemius muscles were lacerated and collected for histological analysis at day seven post-injury. (**A**) Representative images of CD31-stained sections (scale bar = 50 µm, 20× magnification); (**B**) Percent area that stained positive for CD31; (**C**) Representative images of F4/80-stained sections (scale bar = 50 µm, 20× magnification); (**D**) Percent area that stained positive for F4/80. Data are presented as mean ± SE. *n* = 6 per group.

Since LIV increased muscle fiber size and tended to reduce fibrosis after 14 days of treatment (Figures 2–4), the experiment was repeated and muscles were harvested at day seven to determine whether LIV induces early improvements in muscle regeneration. After seven days of treatment, LIV at 90 Hz/0.2 g did not noticeably improve the healing of lacerated gastrocnemius muscle (Figure 5A,B). Compared to non-LIV but injured control mice, both the minimum fiber diameter and the cross-sectional area of individual myofibers were not different in mice treated with LIV at 90 Hz/0.2 g on day seven post-injury (Figure 5C,D). When centrally-nucleated and peripherally-nucleated myofibers were assessed separately, neither minimum fiber diameter, fiber area, nor morphological characteristics were different between treatment groups (Figure 5E–G). Additionally, no differences were found in markers for angiogenesis or macrophage accumulation, as assessed histologically by staining with CD31 and F4/80, respectively (Figure 6). Taken together, these data indicate that LIV does not influence the early regenerative phase of healing and instead improves healing through an influence on the remodeling phase. Alternatively, seven days of LIV treatment may not be sufficient to induce observable improvements in the healing process.

### 3.5. Effects of LIV on Uninjured Muscle

**Figure 7:**
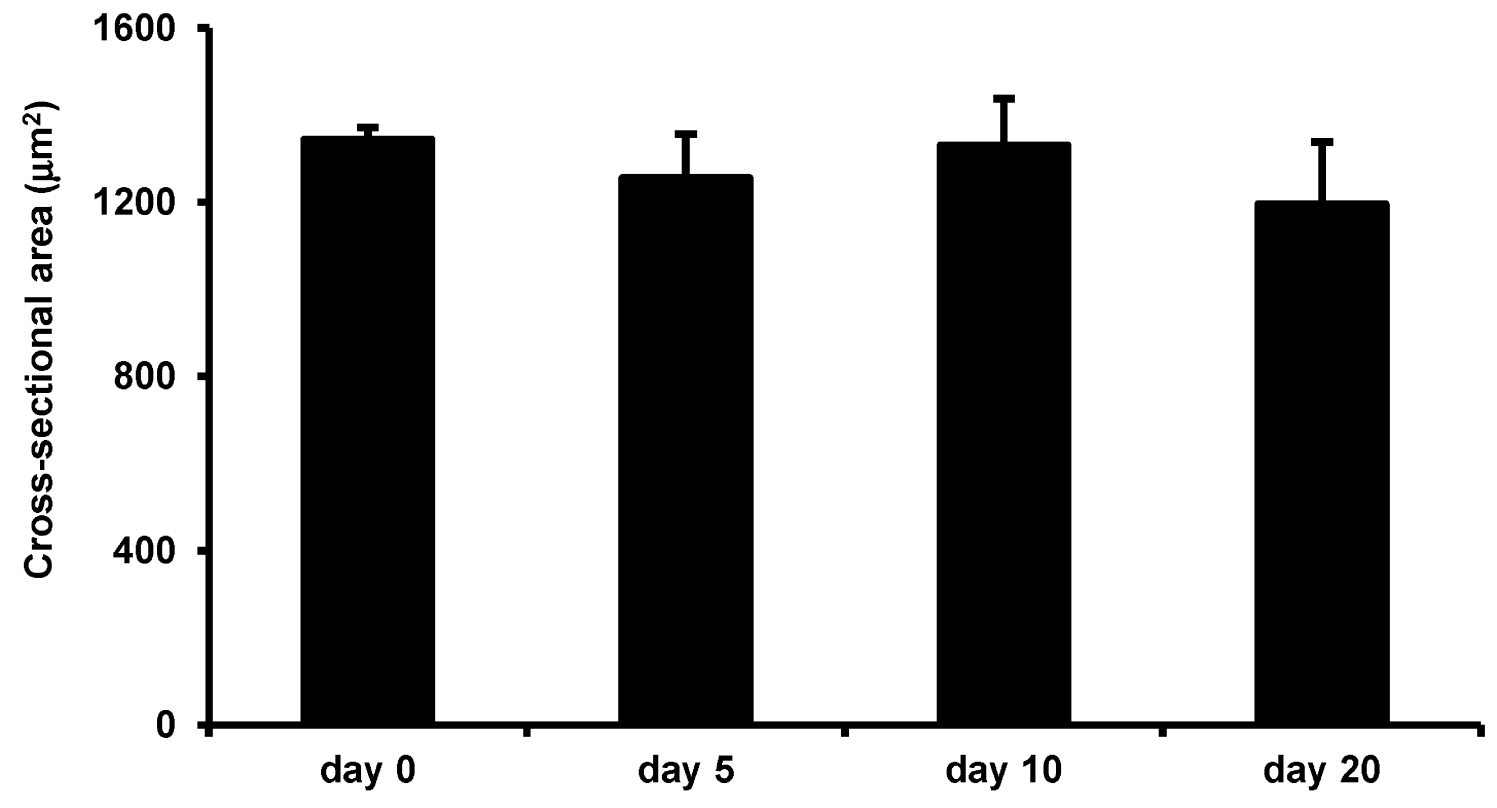
Low-intensity vibration does not enhance myofiber size in uninjured mice. Uninjured control mice were subjected to the LIV protocol and gastrocnemius muscles were collected at the indicated time points. Average cross-sectional area of individual myofibers was quantified in five 40× fields per muscle in hematoxylin and eosin-stained sections. Data are presented as mean ± SE.

Interestingly, in uninjured mice, LIV did not increase the average myofiber cross-sectional area after 20 days of LIV treatments, suggesting that the beneficial effects of this LIV protocol do not accrue to non-injured skeletal muscle, but likely require prior muscle damage and subsequent regeneration (Figure 7).

## 4. Discussion

Unlike toxin- or exercise-induced muscle injuries, recovery from traumatic muscle injuries is typically prolonged and incomplete [1, 25, 26], resulting in permanent impairments of muscle and joint function [2–5]. This impaired healing results in significant costs for rehabilitation, loss of time for work, and reduced combat readiness in military personnel [25]. Thus, effective therapies for promoting the healing of traumatic muscle injuries are needed. Interestingly, mechanical stimulation via LIV has been shown to ameliorate bone loss and to enhance bone regeneration [16–19]. Furthermore, we have recently shown that LIV improves the delayed healing of skin wounds in diabetic mice [21]. However, little is known about the effects of LIV on the healing of damaged muscle. We therefore determined whether mechanical stimulation via LIV could (1) improve muscle regeneration and (2) reduce muscle fibrosis following traumatic injury in mice. Our findings provide evidence that LIV indeed improves muscle repair by influencing the remodeling phase of healing.

To our knowledge, this is the first study to assess the effects of mechanical stimulation via LIV on muscle healing. We observed a larger myofiber size in mice that received LIV treatment protocols at 90 Hz/0.2 g or 45 Hz/0.4 g for 14 days post-injury, but not for seven days post-injury, compared to non-LIV controls. LIV did not promote myofiber hypertrophy after 20 days of LIV treatments in uninjured mice. These findings indicate that prior muscle damage and subsequent regeneration is likely required for the beneficial effects of LIV. While the pathways that modulate the cellular response to LIV remain to be elucidated, we can speculate that LIV may exert local and/or systemic effects and that these effects are likely at later stages of healing since improvements were not seen until 14 days post-injury. LIV may increase fiber size via direct mechanical effects on muscle cells, since muscle is particularly sensitive to mechanical stimuli, or indirectly via the production of cytokines and growth factors that promote muscle growth. Alternatively, it is well documented that LIV can be anabolic to bone, and thus, LIV may promote the mobilization and/or homing of bone marrow-derived cells to the injured tissue. These cells include progenitor cells and monocytes/macrophages, which are important during tissue repair as they release growth factors and cytokines that promote tissue healing [6, 7, 10, 27]. Our findings suggest that LIV may have anabolic effects on regenerating muscle and the elucidating mechanisms underlying the local and/or systemic effects of LIV warrant further investigation.

The development of fibrosis likely contributes to the impaired healing of traumatic muscle injuries. As such, experimental therapeutic approaches have attempted to improve healing by blocking actions of transforming growth factor (TGF)-β1 and the associated fibrosis; these antifibrotic agents have included suramin, interferon (IFN)-γ, decorin, and losartan [28–31]. While these agents have shown promise in animal studies in reducing fibrosis and improving regeneration following traumatic muscle injury, many of these agents have serious side effects and would likely not be an option for treating muscle injuries. In the current study, the trend of reduced collagen deposition following injury in the LIV-treated mice at 14 days post-injury with the 45 Hz protocol suggests that LIV may serve as a safe, non-pharmacological therapy for reducing fibrosis. Because LIV was initiated within hours of the injury, our findings suggest that LIV may be effective in attenuating or preventing fibrosis. Whether or not LIV can reverse fibrosis after it has been established warrants further investigation.

One reason that healing is impaired in models of traumatic injury (such as laceration) compared to other injury models (such as toxin-induced injury) may be the disruption of blood supply to the muscle. Thus, improving the perfusion of damaged muscle may be an additional mechanism by which LIV can improve healing. Although LIV did not improve CD31 staining, a marker for angiogenesis, at day seven in the current study, we have recently shown that LIV improves the delayed healing of skin wounds in diabetic mice, which was associated with an increase in CD31 staining [21]. LIV can also acutely increase blood flow in the skin of the ear of hairless mice, the skin of the dorsal side of the lower leg of healthy human subjects, and the skin of the underside of the forearm of both healthy and Type 2 diabetic human subjects [32–34]. Furthermore, nitric oxide (NO) is well-known for its vasodilatory effects. Serum nitrite levels, a marker for NO signaling, increases with the application of LIV in juvenile pigs [35, 36]. L-NAME, an NO synthase inhibitor, blocked the LIV induced increase in skin blood flow in the ear of hairless mice [32, 35, 36]. LIV has been shown to improve the healing of pressure ulcers in humans by upregulating NO and improving blood supply [37]. Relatedly, LIV also slowed the progression of pressure ulcers into deep tissue injury in a rat model [38]. Thus, future studies should further investigate the influence of LIV on blood vessel formation and the perfusion of damaged skeletal muscle.

Our study is limited in that the effects of LIV on muscle healing were only assessed at two time points. Skeletal muscle repair following injury occurs in four overlapping phases: hemostasis, inflammation, new muscle fiber formation, and subsequent remodeling. Our findings are likely relevant to the remodeling phase as we have previously shown that new myofiber formation predominates during the first two weeks following muscle laceration, while myofiber maturation and collagen deposition typically occur thereafter [22]. We are currently performing a time course study that investigates the effect of LIV on muscle healing during each of the different phases of healing. This study is also limited in that mechanisms underlying LIV-induced improvements in healing were not thoroughly investigated. The purposes of this initial study were to evaluate whether or not whole-body LIV could be a feasible and effective strategy for improving the healing of a traumatic muscle injury and whether varying LIV parameters (frequency and amplitude) had an impact on improving the healing of a traumatic muscle injury. Further optimization of the LIV protocol may yield even better results. Now that we have established LIV as a potential therapeutic strategy for muscle healing, mechanistic studies are ongoing. Finally, this study is limited by the lack of assessment of muscle functional recovery. We plan to determine the effect of LIV on the time course of functional recovery in a future study.

In summary, our findings are consistent with our hypothesis that LIV improves muscle regeneration and reduces fibrosis following traumatic injury. Thus, LIV may provide a novel, non-pharmacological therapeutic approach for improving the prolonged and incomplete healing typically seen with these injuries. The LIV protocol used in this study is simple, inexpensive, and safe; at the amplitude employed (<100 µm), the vibration is barely perceptible to human touch. Furthermore, the LIV protocol could be easily translated to use for human studies, since the equipment utilized has already been used to ameliorate bone loss in human subjects [17, 39–42].
